# Selective targeting of the BRG/PB1 bromodomains impairs embryonic and trophoblast stem cell maintenance

**DOI:** 10.1126/sciadv.1500723

**Published:** 2015-11-13

**Authors:** Oleg Fedorov, Josefina Castex, Cynthia Tallant, Dafydd R. Owen, Sarah Martin, Matteo Aldeghi, Octovia Monteiro, Panagis Filippakopoulos, Sarah Picaud, John D. Trzupek, Brian S. Gerstenberger, Chas Bountra, Dominica Willmann, Christopher Wells, Martin Philpott, Catherine Rogers, Philip C. Biggin, Paul E. Brennan, Mark E. Bunnage, Roland Schüle, Thomas Günther, Stefan Knapp, Susanne Müller

**Affiliations:** 1 Target Discovery Institute, University of Oxford, NDM Research Building, Roosevelt Drive, Oxford OX3 7FZ, UK.; 2 Structural Genomics Consortium, University of Oxford, Old Road Campus Research Building, Roosevelt Drive, Oxford OX3 7DQ, UK.; 3 Urologische Klinik und Zentrale Klinische Forschung, Klinikum der Universität Freiburg, Breisacher Strasse 66, 79106 Freiburg, Germany.; 4 Pfizer Worldwide Medicinal Chemistry, 610 Main Street, Cambridge, MA 02139, USA.; 5 Department of Biochemistry, University of Oxford, South Parks Road, Oxford OX1 3QU, UK.; 6 Ludwig Institute for Cancer Research, University of Oxford, Oxford OX3 7DQ, UK.; 7 Deutsches Konsortium für Translationale Krebsforschung, Standort Freiburg, 79106 Freiburg, Germany.; 8 BIOSS Centre of Biological Signalling Studies, Albert-Ludwigs-University Freiburg, 79106 Freiburg, Germany.; 9 Institute for Pharmaceutical Chemistry and Buchmann Institute for Molecular Life Sciences, Johann Wolfgang Goethe-University, Max-von-Laue-Str. 9, D-60438 Frankfurt am Main, Germany.

**Keywords:** epigenetics, chromatin remodelling,embryonic stem cells, trophoblast stem cells, BRG, BRM, PB1, chemical probe, BAF complex

## Abstract

Mammalian SWI/SNF [also called Brg/Brahma-associated factors (BAFs)] are evolutionarily conserved chromatin-remodeling complexes regulating gene transcription programs during development and stem cell differentiation. BAF complexes contain an ATP (adenosine 5′-triphosphate)–driven remodeling enzyme (either BRG1 or BRM) and multiple protein interaction domains including bromodomains, an evolutionary conserved acetyl lysine–dependent protein interaction motif that recruits transcriptional regulators to acetylated chromatin. We report a potent and cell active protein interaction inhibitor, PFI-3, that selectively binds to essential BAF bromodomains. The high specificity of PFI-3 was achieved on the basis of a novel binding mode of a salicylic acid head group that led to the replacement of water molecules typically maintained in other bromodomain inhibitor complexes. We show that exposure of embryonic stem cells to PFI-3 led to deprivation of stemness and deregulated lineage specification. Furthermore, differentiation of trophoblast stem cells in the presence of PFI-3 was markedly enhanced. The data present a key function of BAF bromodomains in stem cell maintenance and differentiation, introducing a novel versatile chemical probe for studies on acetylation-dependent cellular processes controlled by BAF remodeling complexes.

## Teaser

PFI-3, a novel inhibitor targeting the bromodomains of essential components of the BAF/PBAF complex, affects the differentiation of ESC and TSC.

## INTRODUCTION

The combinatorial assembly of alternative Brahma-associated factor (BAF) family members creates a diverse family of cell type–specific remodelling complexes that exert context-dependent functions. Comparison with simple eukaryotes such as yeast showed that the central core of five orthologs (BRG1/BRM, BAF155/170, BAF60, BAF53a/b, and BAF47) is evolutionarily conserved. In mammals, several additional subunits are present (BAF250a/BAF250b, BAF200, BAF45a/b/c/d, BRD9/BRD7, and BAF57), which may be supplemented with cell type–specific BAF subunits that regulate tissue-specific gene transcription programs (*1*, *2*). For example, distinct BAF complexes [polybromo-BAF (PBAF)] that harbor an additional subunit BAF180 [polybromo-1 (PB1)] have been identified in cardiac progenitors (*3*). A specific BAF complex, esBAF, present in embryonic stem cells (ESCs) exclusively contains the BRG1 (SMARCA4) and not the related BRM (SMARCA2) catalytic subunit (*4*, *5*). Genetic deletion of BRG1 and other BAF core subunits revealed that BAF complexes are required not only for ESC maintenance but also for proper lineage specification (*4*–*8*). Knockdown of Brg1 initially increased expression of ESC marker genes such as Oct4, whereas transcription was decreased at later time points (*9*). Furthermore, development of the trophectoderm, which gives rise to the embryonic part of the placenta, crucially depends on members of the BAF complex, whereas the genesis of trophoblast stem cells (TSCs) seems not to be affected (*6*–*8*).

BRG1 harbors a C-terminal bromodomain (BRD), which is evolutionarily highly conserved (fig. S1). BRDs were first identified as a conserved sequence motif present in the *Drosophila* homolog of BRM (*10*), and they constitute a family of 61 highly diverse interaction modules present in 46 proteins in humans (*11*). BRDs selectively recognize ε-*N*-lysine acetylation motifs, a key interaction in the reading process of posttranslational modifications that constitute the epigenetic code. The recent discovery of potent and highly specific inhibitors for the BET family of BRDs (*12*–*14*) has stimulated intensive research activity, particularly in oncology where BET proteins regulate the expression of key oncogenes, with the first BET inhibitors recently entering clinical testing (*15*, *16*). The predicted favorable “druggability” of these protein interaction domains (*17*) suggested that other BRD family members can also be selectively targeted.

## RESULTS

### Initial inhibitor hit with unusual binding mode

BAF complexes contain several BRD-containing proteins (BRG1/BRM, PB1, BRD7, and BRD9). Deletion studies in flies, however, indicated that not all BAF BRDs are required during development (*18*). However, because of the presence of multiple BRDs in BAF, which may exert compensatory functions, the consequences of BRD inhibition in a certain cell type cannot be easily predicted by genetic knockout studies. To study the role of BAF bromodomains in chromatin biology, as well as to explore opportunities for the development of new pharmacological strategies, we developed a selective chemical tool compound targeting the highly related BRDs of BRG1/BRM.

**Fig. 1:**
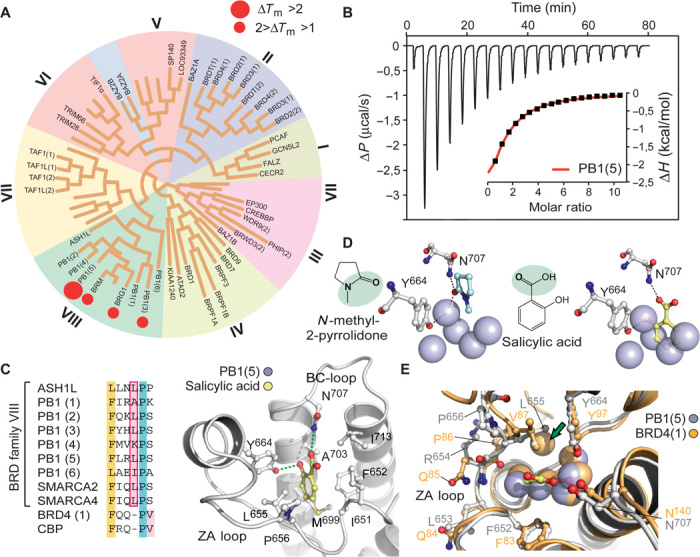
Characterization of the interaction of SA with family VIII bromodomains. (**A**) Selectivity of SA assessed using temperature shift (Δ*T*_m_) assays. Screened targets are labeled in the phylogenetic tree based on the BRD family structure–based alignment (*11*). (**B**) Isothermal titration data of the interaction of SA with PB1(5). The raw binding heats for each injection as well as the normalized binding enthalpies (inset) are shown. (**C**). Sequence alignment of a ZA-loop segment of bromodomains of family VIII as well as BRD4(1) and CBP (CREB-binding protein) (left panel) and structural overview of the SA/PB1(5) complex. Hydrogen bonds to the conserved asparagine (N^707^) and tyrosine (Y^664^) are shown as dotted green lines. (**D**) Binding mode of a typical acetyl lysine mimetic fragment (*N*-methyl-2-pyrrolidone) in PB1(5) (left panel) and SA (right panel). Water molecules are shown as solid spheres. Waters present in the PB1(5) complex are superimposed onto the SA cocrystal structure to demonstrate the displacement of four structural waters by the ligand. (**E**) Superposition of apo-PB1(5) (PDB ID: 3G0J) and apo-BRD4(1) (PDB ID: 2oss); blue spheres represent waters from the PB1(5)/NMP structure. There is a missing water indicated with a green arrow for PB1(5) structures.

Initial screening of chemical libraries containing acetyl lysine mimetic ligands failed to identify any significant interaction with BAF BRDs. Additionally, promiscuous BRD inhibitors such as bromosporine and the related [1,2,4]triazolo[4,3-a]phthalazines (*19*) also did not exhibit any binding. Intriguingly, screening of a set of drug-like fragments identified salicylic acid (SA) as a potential starting point, revealing good selectivity for BRDs present in BRG1/BRM and PB1 when profiled against 48 BRDs using a thermal shift assay (*20*) (Fig. 1A and table S1). SA is known to inhibit the transcription of COX-2 resulting in anti-inflammatory activity, and it is the bioactive compound of the prodrug aspirin (*21*). Binding of SA was confirmed in solution using isothermal titration calorimetry (ITC) with the fifth bromodomain of PB1 [PB1(5)], the BAF bromodomain exhibiting the highest temperature shift (Fig. 1B and table S2). ITC data yielded a *K*_D_ (dissociation constant) value of 250 μM, and considering the low molecular weight of SA, the binding data suggested excellent ligand efficiency of this fragment. To obtain insight into the binding mode of SA with PB1(5) and the excellent selectivity of this small fragment toward family VIII BRDs, we determined its cocrystal structure with PB1(5) (table S3). The structure revealed an acetyl lysine mimetic binding pose of SA in which the ligand formed the canonical hydrogen bond with the conserved asparagine (N^707^) (Fig. 1C), which typically anchors the acetyl lysine carbonyl to the BRD binding cavity (*22*). A second hydrogen bond was formed with Y^664^, a conserved residue that usually forms a water-mediated bridge to the acetylated lysine. The binding of SA was further stabilized by van der Waals interactions with L^655^, a residue that is uniquely conserved in PB1(2), PB1(5), BRG1, and BRM (Fig. 1C) (*11*), suggesting that this unique interaction confers the high selectivity of SA toward this subset of BRDs. Comparison with other BRD-peptide and BRD-inhibitor complexes revealed the SA bound deeper into the acetyl lysine binding pocket, leading to an unprecedented displacement of four highly conserved water molecules (Fig. 1D and fig. S2). Comparison with the structure of BRD4(1) (*11*) revealed high positional conservation and similar interactions of the conserved binding site water molecules when compared with the apo structure of PB1(5) [Protein Data Bank (PDB): 3G0J] (Fig. 1E). Intrigued by this novel binding mode, we studied the cavity hydration properties (*23*) and calculated differences in water-protein interaction energies between PB1(5) and BRD4(1) to identify potential structural reasons for the diverse water and ligand-binding behavior of PB1/BRG/BRM BRDs, highlighting a significant energetic difference for the backbone-water interaction of R^654^ (Q^85^ in BRD4), responsible for weakening the water network in PB1(5) (fig. S3).

### Development and in vitro characterization of PFI-3

**Fig. 2:**
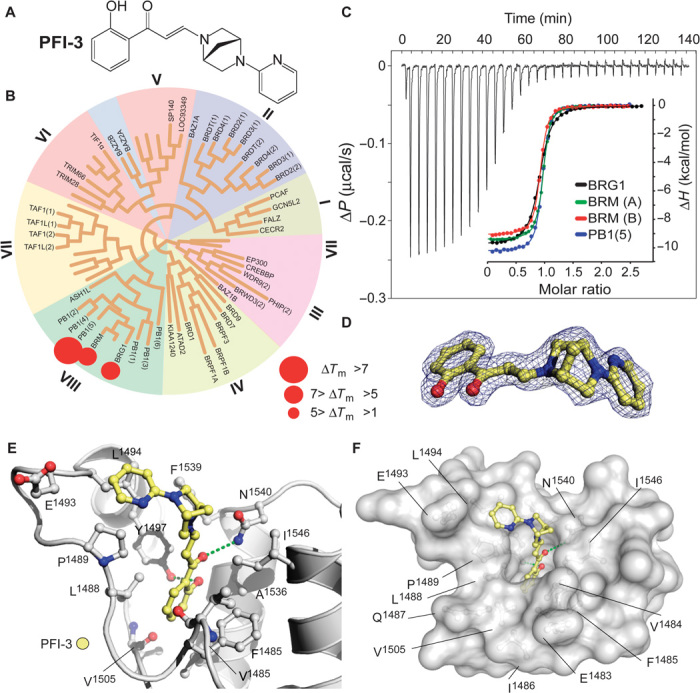
Selectivity, potency, and cocrystal structure of PFI-3 with BRG1. (**A**) Chemical structure of PFI-3. (**B**) Selectivity screening data of PFI-3 using temperature shift assay. The temperature shifts are mapped onto the phylogenetic tree using red spheres as indicated in the figure. (**C**) ITC data measured for PFI-3 using the two BRM bromodomain isoforms (see fig. S1) BRG1 and PB1(5). Raw injection heats as well as normalized binding enthalpies and the corresponding nonlinear least squares fits (inset) are shown. (**D**) 2*F*_o_ − *F*_c_ omit electron density map contoured at 1.5 σ around the inhibitor at 2. (**E**) Details of the interaction of PFI-3 with the BRG1 bromodomain. (**F**) Surface representation of the BRG1 acetyl lysine binding site in complex with PFI-3.

The unique binding mode and good ligand efficiency of SA presented an ideal starting point for the structure-guided design of more potent inhibitors for a subset of BAF BRDs. Several cycles of medicinal chemistry optimization, screening, and cocrystallization yielded the final chemical probe compound PFI-3 (Fig. 2A and table S3). The synthetic efforts and the associated structure-activity relationship of the synthesized compounds will be reported elsewhere. Selectivity screening against 48 members of the human BRD family confirmed the preservation of this chemotype’s selectivity toward BRDs of family VIII, using the same head group identified in SA. Significant Δ*T*_m_ shifts were only detected for BRDs of BRG1/BRM and PB1(5), but not PB1(2), indicating excellent selectivity (Fig. 2B). PFI-3 was additionally screened in a commercial screening panel comprising 102 cellular receptors as well as 30 enzyme assays (CEREP, http://cerep.fr), revealing only interactions against four GPCRs (G protein–coupled receptor) with micromolar affinity and no significant additional interactions outside the BRD family, confirming its excellent broader pharmacological selectivity (table S4). It also did not intercalate into DNA as shown by DNA unwinding assays (fig. S4). Binding constants of PFI-3 with BRDs identified as positive in Δ*T*_m_ screening were determined in solution using ITC, revealing *K*_D_ values between 54 and 97 nM (Fig. 2C and table S2). Dose-dependent biolayer interferometry (BLI) measurements yielded similar *K*_D_ values (fig. S5). We also generated an inactive control compound in which a methoxy group blocked access to the bromodomain acetyl lysine binding site. This compound (PFI-3oMet) showed no binding to BAF bromodomains in temperature shift assays and can therefore be used as a control compound in phenotypic cell assays (fig. S6). The cocrystal structure of PFI-3 with the BRD of BRG1 revealed excellent shape complementarity with the acetyl lysine binding site. In addition, interactions observed in the SA complex with PB1(5) were fully conserved, whereas the bridged saturated ring system, introduced in PFI-3 to sterically protect the hydrolysis-prone enamine bond, resulted in a sharp kink positioning of the pyridine ring within a hydrophobic surface pocket created by the ZA-loop (Fig. 2, D to F). Stability measurements confirmed that PFI-3 has a half-life in aqueous solutions exceeding 7 days at 37°C.

### Cellular activity of PFI-3 and its effect on early development

**Fig. 3:**
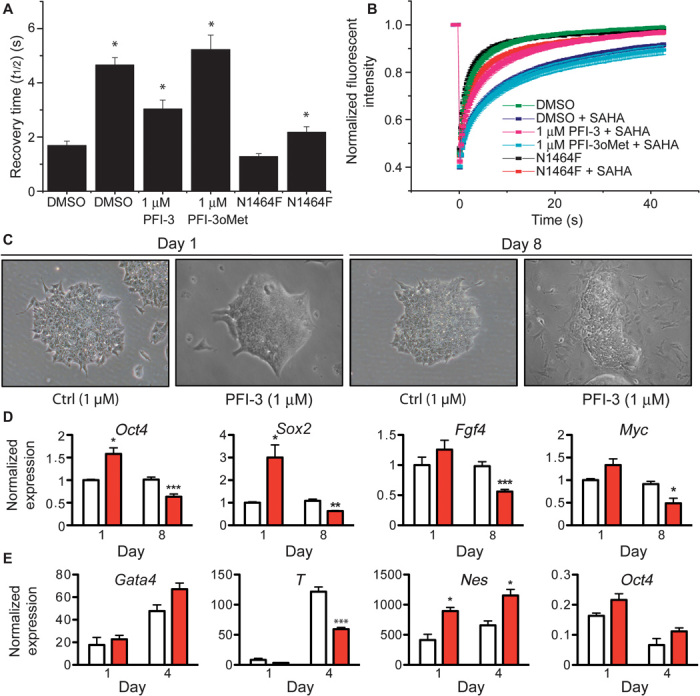
FRAP experiments and loss of stemness, and skewed differentiation in embryonic stem cells (ESCs). (**A**) Influence of PFI-3 or the inactive PFI-3oMet on half recovery times of U2OS cells transfected with wild-type (WT) full-length GFP-BRM or the N1464F mutant construct. Cells were treated with 2.5 μM SAHA (shown by “*”) to increase the assay window. (**B**) Time dependence of fluorescence recovery in the bleached area of cells expressing WT or mutant GFP-BRM with the corresponding treatment as in (A). All PFI-3–treated samples showed a *P* < 0.0001 compared to WT treated with SAHA. Curves represent averaged data of at least 10 replicates. (**C**) Phase-contrast microscopy images of ESCs treated with the negative control substance PFI-3oMet or PFI-3. (**D**) qRT-PCR analyses of the transcription levels for the indicated stemness genes after treatment for 1 or 8 days. (E) mRNA levels of epiblast differentiation genes, representative of endoderm (*Gata4*), mesoderm (*T*), and ectoderm (Nes) cultured for 1 or 4 days. The stemness marker *Oct4* was used as differentiation control. (D and E) mRNA levels normalized to *Gapdh*, *Hprt*, and *Ppia* are represented as means + SEM relative to the expression in ESCs treated with control compound for 24 hours. Experiments were independently repeated at least three times in triplicate. *P < 0.05; ***P < 0.001. ESCs were treated with the negative control substance PFI-3oMet or PFI-3 inhibitor in the presence (C and D) or absence (E) of leukemia inhibitory factor (LIF) in stemness or differentiation conditions, respectively. Red bars represent PFI-3–treated cells, and white bars represent the corresponding control experiments.

To test PFI-3’s ability to displace BRM from chromatin, we performed a fluorescence recovery after photobleaching (FRAP) assay (*24*). U2OS cells transfected with full-length green fluorescent protein (GFP)–tagged BRM were treated with the histone deacetylase inhibitor suberoylanilide hydroxamic acid (SAHA) for 1 hour to increase the assay window and were subsequently exposed to PFI-3 or the inactive control compound PFI-3oMet for 1 hour. PFI-3, but not PFI-3oMet, significantly accelerated the half-recovery time of the wild-type protein when compared to the N1464F mutant that is unable to bind to acetylated chromatin (Fig. 3, A and B). After exposure of the cells for 24 hours, PFI-3 did not show any loss in its ability to displace BRM from chromatin, indicating good chemical stability at 37°C for 24 hours (fig. S7).

Because BRG/BRM have been implicated in tumorigenesis (*25*), we tested the effect of PFI-3 on cell proliferation and survival using the NCI-60 panel of tumor cell lines (data not shown). Exposure of cells to 10 μM inhibitor did not result in significant toxicity for any of the cell lines tested, indicating that the BRD is not important for tumor cell proliferation and survival, as also suggested by a recent study on the role of BRG1 in leukemia (*26*).

BAF complexes are crucial for the biology of ESC and TSC (*6*–*8*), and therefore, we next investigated the consequences of BAF bromodomain inhibition by PFI-3. Culture of ESCs in the presence of 1 μM PFI-3 led to visible differentiation in phase-contrast micrographs after 8 days, in comparison to ESC treated with the negative control substance PFI-3oMet notwithstanding stemness-maintaining conditions (Fig. 3C). In agreement, quantification of transcription after 1 and 8 days of culture in the presence of the inhibitor reflected the documented initial up- and subsequent down-regulation of stemness genes (Fig. 3D) after knockdown of Brg1 (*9*). We also examined the impact of the PFI-3 inhibitor on the differentiation of ESCs comparing gene expression levels of key differentiation genes of endoderm (*Gata4*), mesoderm (*T*, brachyury homolog), and ectoderm (*Nes*) by quantitative reverse transcription polymerase chain reaction (qRT-PCR) analyses. *Nes* expression was increased after 4 days of differentiation in the presence of PFI-3 compared to treatment with a control compound at the expense of the brachyury homolog T transcription, whereas *Gata4* expression was not significantly altered. Differentiation of ESCs was verified by the decrease in *Oct4* levels (Fig. 3E). Furthermore, we compared the impact of PFI-3 to the reported effect of Brg1 deletion on the repressive chromatin mark H3K27me3 (trimethylated lysine 27 of histone 3) in ESCs by chromatin immunoprecipitation (ChIP) (*27*). We first verified the repression of known Brg1 and Stat3 target gene transcription by qRT-PCR in ESCs treated with either PFI-3 or the inactive control inhibitor PFI-3oMet (fig. S8A). Similar to the data reported by Ho *et al.* (*27*), we observe an increase of H3K27me3 at common Brg1 and Stat3 binding sites at promoters and at transcription start sites (TSSs; fig. S8, B and C) consistent with gene repression.

**Fig. 4:**
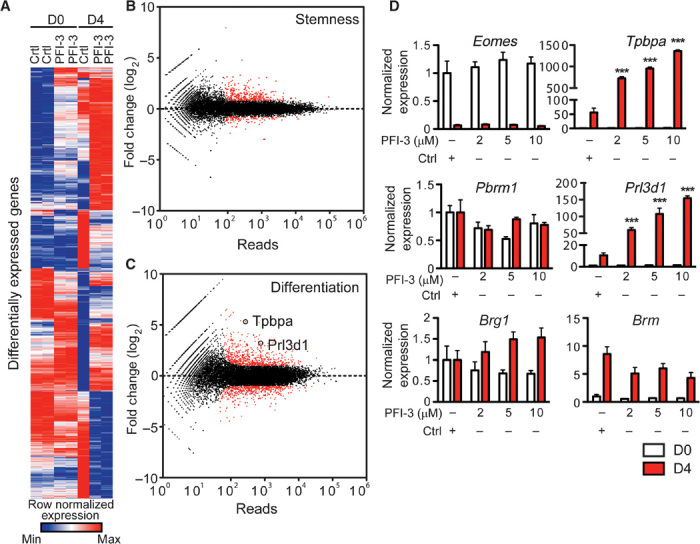
TSC differentiation is affected by PFI-3 inhibition. Comparison of gene expression in TSCs treated with PFI-3 or control substance during stemness and differentiation. (**A** to **C**) Differentially transcribed genes are displayed normalized and clustered to the number of reads shown as (A) tile array and (B and C) dot blots. (**D**) Quantification of stemness (*Eomes*) and differentiation markers (*Prl3d1*, Tpbpa) expression by RT-PCR. mRNA levels were normalized to *Gapdh*, *Hprt*, and *Ppia*, and values represent the means + SEM relative to the expression in TSCs treated with control compound at day 0. Experiments were independently repeated at least three times in triplicate. ***P < 0.001.

Trophoblasts are the first cells to differentiate in the fertilized egg forming the outer layer of the blastocyst. To further evaluate the function of the targeted BAF bromodomains in stem cells, we treated TSC with 2 μM inhibitor or control substance and related global gene expression by RNA sequencing under stemness (D0) and differentiation (D4) conditions (Fig. 4, A to C, and table S5). Gene set enrichment analyses implicated insulin signaling and mRNA processing as the most significantly regulated pathways for stemness and differentiation, respectively, in the presence of the inhibitor (fig. S9). Expression levels were verified by qRT-PCR, confirming a marked increase of key TSC differentiation markers (*Prl3d1* and *Tpbpa*) (Fig. 4D). Enhanced *Prl7d1* and *Tpbpa* transcription could still be observed after washout of the inhibitor during differentiation of TSCs albeit to a lesser extent (fig. S10A). Differentiation of TSC was verified by decrease in transcription of the stemness gene *Eomes*. Levels of *Eomes*, *Brg1*, *Brm*, and *Pbrm1/Baf180* were not affected by the PFI-3 inhibitor (Fig. 4D). We verified the specificity of PFI-3 in TSCs by quantification of gene expression after knockdown of *Brm* and *Brg1* in stemness and differentiation conditions (fig. S10, B and C). Together, our data demonstrate that inhibition of the Brg1 and Brm BRDs in BAF critically impairs the function of ESCs and TSCs.

## DISCUSSION

Here, we developed a highly potent, selective, and cell active inhibitor for the BAF BRDs BRG1/BRM and PB1(5) and studied the consequences of inhibiting these protein interaction modules in ESCs and TSCs. The developed inhibitor PFI-3 displayed a novel binding mode for BRD inhibitors and provides a versatile tool for studying acetyl lysine–dependent cellular function of the targeted BRDs, offering the potential for targeting these protein interaction domains for the development of new treatment strategies. Unexpectedly, the inhibitor was well tolerated and did not cause toxicity after short time exposure. In long-term experiments, PFI-3 significantly altered gene expression programs that are important for differentiation of stem cells. We demonstrate that the bromodomains of Brg1 and Brm are critical for the function of ESCs and TSCs, exemplified by the modulation of gene transcriptional programs regulating cell differentiation. This is contrary to observations made in flies, where the bromodomain of BRM seems dispensable for the function of the protein (*18*). Despite conversation of these genes, it seems that Brm and Brg1 have acquired diverse function in higher eukaryotes critical for development and cell differentiation. Similar conclusions have also been made by Ho *et al*. (*27*), which observed conserved as well as new functions of the BAF complex in vertebrates. For the studied Brg1 and Stat target genes, we observed that PFI-3 mimics the effect of Brg1 deletion, resulting in an increase of the repressive H3K27me3 mark at the TSSs and Stat3 binding sites of these genes.

Long delays in the response to compound treatment have also been observed for other inhibitors that target chromatin organization such as methyl lysine transferase inhibitors (*28*). The slow onset of drug action complicates drug development that currently relies on the immediate response of inhibitors on biomarkers to monitor drug efficacy and potency in cellular systems and in vivo. Slow changes of the organization of the epigenome may generate long-lasting effects on gene expression programs that control cell function, which may not be detected in classical toxicology studies. In the case discussed here, the prolonged use of high concentration of aspirin may cause long-lasting epigenetic changes by inhibiting BAF bromodomains.

BAF complexes play an important role in many differentiation processes, and mutations in their components are dominant drivers in cancer (*25*), resulting in a considerable interest in the role of BAF chromatin remodeling in tumorigenesis. Large-scale exon sequencing revealed that about 20% of human tumors carry mutations in subunits of BAF complexes. Mutations typically lead to loss of function, suggesting tumor suppressor roles of BAF complexes, in particular in childhood malignant rhabdoid tumors (loss of SMARCB1/SNF5) (*29*) as well as ovarian and endometrial carcinomas (ARIAD1A) (*30*, *31*). Additionally, most renal cell carcinomas have a dysfunctional PB1 protein (*32*); however, a recent study indicated that the ATPase (adenosine triphosphatase) domain and not the BRD is essential for cell proliferation and survival, at least in hematopoietic malignancies (*26*). Our data point toward a crucial role of the BRD during differentiation of stem cells, suggesting that in cancer, targeting of BRG1/BRM BRDs may mainly affect cancer stem cell maintenance rather than causing immediate cytotoxicity on tumor cells. PFI-3 represents a versatile tool for studying the role of the targeted BAF bromodomains in cancer as well as in other cellular systems in the future.

## MATERIALS AND METHODS

### Protein purification

Complementary DNA–encoding human bromodomains were cloned, expressed, and purified as previously described (*11*). For purification of in vivo biotinylated protein expression, the same construct boundaries [for example, CBP residues R1081-G1198] were bromodomain subcloned into pNIC-BIO1 vector, a derivative from pNIC28-Bsa4 vector (GenBank: EF198106), containing a 10 His-tag and TEV protease cleavage site at the N terminus and an in-frame biotinylation sequence (SSKGGYGLNDIFEAQKIEWHE) inserted at the C terminus. The constructs were transformed into BL21 (DE3)-R3-BirA cell line (BL21 derivative coexpressing BirA using a pACYC coexpression vector). Cells were grown overnight at 37°C in 10 ml of Luria-Bertani medium with kanamycin (50 μg/ml) and chloramphenicol (34 μg/ml) (startup culture). The startup culture was diluted 1:1000 in fresh medium, and cell growth was allowed at 37°C to an optical density of about ~1.0 (OD_600_) before the temperature was decreased to 25°C. d-Biotin was dissolved into 10 mM bicine (pH 8.3) and added to the culture at 500 μM final. The protein expression was induced for 8 hours at 25°C with 50 μM isopropyl-β-d-thiogalactopyranoside (IPTG). Proteins were purified using Ni affinity chromatography and size exclusion chromatography.

### Biolayer interferometry

Kinetic ligand-binding measurements were done using an Octet RED384 instrument (ForteBio). Biotinylated protein was immobilized on Super Streptavidin Biosensors using a concentration of 0.05 mg/ml. Association and dissociation measurements were done in 25 mM Hepes (pH 7.4), 100 mM NaCl, 0.01% Tween at 25°C with association and dissociation times of 240 s. Compounds were prepared as 1:2.5 dilutions starting from 12 μM. Binding to the reference sensors (no protein attached) was subtracted before calculations. Binding constants were calculated using the ForteBio Analysis software provided by the manufacturer.

### Isothermal titration calorimetry

Experiments were carried out on a VP-ITC microcalorimeter (MicroCal). All experiments were performed at 15°C in 50 mM Hepes (pH 7.5), 150 mM NaCl. The titrations were conducted using an initial injection of 2 μl followed by 34 identical injections of 8 μl. The dilution heats were measured on separate experiments and were subtracted from the titration data. Thermodynamic parameters were calculated using Δ*G* = Δ*H* − *T*Δ*S* = −*RT*ln*K*_B_, where Δ*G*, Δ*H*, and Δ*S* are the changes in free energy, enthalpy, and entropy of binding, respectively. In all cases, a single binding site model was used.

### Thermal shift assay

Thermal melting experiments were carried out using an Mx3005P Real-Time PCR machine (Stratagene). Proteins were buffered in 10 mM Hepes (pH 7.5), 500 mM NaCl, and assayed in a 96-well plate at a final concentration of 2 μM in 20-μl volume. Compounds were added at a final concentration of 10 μM. SYPRO Orange (Molecular Probes) was added as a fluorescence probe at a dilution of 1:1000. Excitation and emission filters for the SYPRO Orange dye were set to 465 and 590 nm, respectively. The temperature was raised with a step of 3°C/min from 25° to 96°C, and fluorescence readings were taken at each interval. Data were analyzed as previously described (*11*, *20*).

### AlphaScreen assay

Assays were performed as described previously (*33*) with minor modifications from the the manufacturer’s protocol (PerkinElmer). All reagents were diluted in 25 mM Hepes, 100 mM NaCl, 0.1% bovine serum albumin (pH 7.4) supplemented with 0.05% CHAPS and allowed to equilibrate to room temperature before addition to plates. An 11-point 1:2.5 serial dilution of the ligands was prepared over the range of 5000 to 0 μM, and 0.1 μl was transferred to low-volume 384-well plates filled with 5 μl of the assay buffer (ProxiPlate-384 Plus, PerkinElmer), followed by 7 μl of biotinylated peptide H-ALREIRRYQK(ac)STELLIRKLK(biotin)-OH and His-tagged protein to achieve a final assay concentration of 50 nM. Plates were sealed and incubated for a further 30 min before the addition of 8 μl of the mixture of streptavidin-coated donor beads (12.5 μg/ml) and nickel chelate acceptor beads (12.5 μg/ml) under low light conditions. Plates were foil-sealed to protect from light, incubated at room temperature for 60 min, and read on a PHERAstar FS plate reader (BMG Labtech) using an AlphaScreen 680 excitation/570 emission filter set. IC_50_ (median inhibitory concentration) values were calculated in Prism 5 (GraphPad Software) after normalization against corresponding dimethyl sulfoxide (DMSO) controls and are given as the final concentration of the compound in the 20-μl reaction volume.

### Fluorescence recovery after photobleaching

FRAP studies were performed using a protocol modified from previous studies (*24*). In brief, U2OS cells were transfected (FuGENE HD, Roche) with mammalian overexpression constructs encoding GFP fused to the N terminus of full-length BRM. The FRAP and imaging system consisted of a Zeiss LSM 710 laser scanning and control system coupled to an inverted Zeiss Axio Observer.Z1 microscope equipped with a high numerical aperture (1.3) 40× oil immersion objective. Samples were placed in an incubator chamber capable of maintaining temperature and humidity. FRAP and GFP fluorescence imaging were both carried out with an argon ion laser (488 nm) and with a photomultiplier tube detector set to detect fluorescence between 500 and 550 nm. Once an initial scan had been taken, a region of interest corresponding to about 50% of the entire GFP-positive nucleus was empirically selected for bleaching. A time-lapse series was then taken to record GFP recovery using 1% of the power used for bleaching. The image data sets and fluorescence recovery data were exported from ZEN 2009, the microscope control software, into Microsoft Excel to determine the average half-time for full recovery for 10 to 20 cells per treatment point.

### Cell culture and reagents

CRG8 ESCs were maintained in serum-containing media supplemented with LIF (1000 U/ml; ESGRO, ESG1106) on feeder-free gelatin-coated wells. ESC culture media contained Dulbecco’s modified Eagle’s medium/knockout (Invitrogen), 15% ES-qualified fetal bovine serum (Invitrogen), 1% penicillin-streptomycin (Lonza, DE17-602E), 1% l-glutamine (Lonza, BE17-605E), 1% sodium pyruvate (Invitrogen, 11360), 1% β-mercaptoethanol, and 1% nonessential amino acids (Invitrogen, 11140). Differentiation of ESCs was induced in the presence of retinoic acid (Sigma, R2625) by withdrawal of LIF supplementation.

Derivation and culture of TSCs were performed as previously described (*34*). Shortly, TSCs were cultured in the absence of feeders in medium supplemented with FGF4 (30 ng/ml; R&D Systems, 235-F4-025/CF), heparin (1.2 μg/ml; Sigma, H3149), and 70% conditional medium (harvested from mitomycin-treated primary mouse embryonic fibroblasts). TSC medium contained RPMI 1640 (BD Biosciences, 354230), 20% ES-qualified fetal bovine serum (Invitrogen), 1% penicillin-streptomycin (Lonza, DE17-602E), 1% l-glutamine (Lonza, BE17-605E), 1% sodium pyruvate (Invitrogen, 11360), and 0.1 mM β-mercaptoethanol. To induce differentiation, TSCs were cultured on feeder-free conditions in TSC medium lacking FGF4 and heparin supplementation.

### qRT-PCR and RNA sequencing

qRT-PCR ESC samples were collected by culturing ESC in stemness conditions in the presence of 1 μM PF1-3 or control compound for 1 or 8 days. ESC-differentiated samples were collected by treating the cells with PF1-3 or control compound 24 hours before differentiation induction. RNA was isolated using RNeasy Mini Kit (Qiagen, 74106) following the manufacturer’s instructions. At least two samples were collected for each group, and each qRT-PCR was run in triplicates. The qRT-PCR data were normalized against the housekeeping genes Gapdh, Ppia, and Hprt and expressed against control compound–treated ESC in stemness conditions. A list of primers is compiled in tables S5 and S6.

To collect TSC samples for qRT-PCR or RNA sequencing (RNA-seq), TSC were cultured in stemness or differentiation conditions and treated with 2 μM PFI-3 or 10 μM control compound or indicated concentrations for 24 hours or during the entire differentiation induction (4 days), respectively. In case of the differentiation samples, the PFI-3 or control compound exposure started 1 day before the induction of differentiation. RNA was isolated as for the ESC samples.

RNA-Seq Library synthesis and analysis were performed at the DKFZ sequencing genomics core facility (Heidelberg, Germany). Mapping and bioinformatics analysis were performed using TopHat, EdgeR Gene-E, and HOMER (*35*–*37*). The raw data from the RNA set results are available through Gene Expression Omnibus (GEO) (accession no. GSE65358). Each replicate represents at least 12 million reads, a density sufficient for qualitative analysis of gene expression. The reads were aligned to the mm10 mouse genome, allowing a mismatch of 2%. We used WebGestalt to find affected pathways (*38*).

### Chromatin immunoprecipitation

ChIP experiments were carried out with Covaris truChIP chromatin shearing reagent according to the manufacturer’s instructions with certain modifications. ESCs were fixed on-plate with 1% formaldehyde in phosphate-buffered saline for 5 min. Cells (10 million) were resuspended in 1 ml of shearing buffer and sonicated (Covaris S220) for 200 cycles per burst, 140 W peak power, 5% duty factor for 15 min in 1-ml Covaris tubes. The supernatant was then diluted to a protein concentration of 1.7 mg ml^−1^, and 350 to 500 μg of protein were used for each immunoprecipitation reaction with anti-H3K27me3 (pab-069-050, Diagenode). The targeted gene fragments were amplified with primers used by Ho *et al.* (*27*).

### RNA interference and Western blot analysis

For RNA interference, TSCs were transfected with Lipofectamine LTX according to the manufacturer’s instructions (Invitrogen). Before transfection, the cells were cultured at least two passages on feeders. Puromycin (1.5 μg/ml; Sigma, P8833) was administrated to the cells 24 hours after transfection. TSCs were transfected with microRNA (miRNA) against mouse Brg1 and Brm1 or LacZ miRNA cloned into pRTS-GW (table S8).

For Western blot analysis, the protein samples were isolated using SC buffer. The following antibodies were used: anti–β-actin (1:15,000, Sigma, A1978), anti-Brg1 (Smarca4) (1:200, Santa Cruz, sc10768), and anti-Smarca2 (Brm) (1:1000, Abcam, ab15597).

### DNA unwinding assay

To establish whether PFI-3 intercalates DNA, the compound was assessed using a DNA unwinding assay, as described by the manufacturer’s protocol (Inspiralis; DUKSR001). In brief, PFI-3 (1, 5, or 10 μM), cisplatin, or doxorubicin was incubated with supercoiled pBR322, in the presence of wheat germ topoisomerase I (TopoI), for 30 min at 37°C. DNA incubated with DMSO (Sigma-Aldrich; D8418) in the presence or absence of the enzyme was run as control. After extraction by butanol (Sigma-Aldrich; 281549) and chloroform/isoamyl alcohol 24:1 (Sigma-Aldrich; C0549), the DNA was run in a 1% (w/v) agarose gel (Web Scientific; AGR-500) with a 1-kb DNA ladder (Life Technologies; 10787) for 4 hours at 80 V. The gel was then stained with SYBR Safe for 30 min before ultraviolet visualization (UVP; BioDoc-It Imaging System).

### Chemistry synthetic methods and compound characterization

Proton (^1^H NMR), carbon (^13^C NMR), and fluorine (^19^F NMR) magnetic resonance spectra were obtained in DMSO-d_6_ at 400, 100, and 376 MHz, respectively, unless otherwise noted. The following abbreviations were used to describe peak patterns when appropriate: br, broad; s, singlet; d, doublet; and m, multiplet. High-resolution mass measurements were obtained on an Agilent time-of-flight mass spectrometer. All air and moisture sensitive reactions were carried out under an atmosphere of dry nitrogen using heat-dried glassware and standard syringe techniques. Tetrahydrofuran (THF) and acetonitrile were purchased from EMD Millipore and were used without further drying. Flash chromatography was performed using an AnaLogix IntelliFlash 280 or Biotage SP1 purification system with Sepra Si 50 silica gel using ethyl acetate/heptane mixtures as solvent unless otherwise indicated. High-performance liquid chromatography was carried out on an Agela Venusil ASB C18 column (21.2 × 150 mm, 5 μm). A flow rate of 0.5 to 150 ml/min was used with mobile phase A: water + 0.1% modifier (v/v) and phase B: acetonitrile + 0.1% modifier (v/v). The modifier was formic acid, trifluoroacetate, ammonia acetate, or hydrochloric acid.

Quality control analysis was performed using a liquid chromatography–mass spectrometry (LCMS) method. Acidic runs were carried out on a Shimadzu XB-C18 (2.1 × 30 mm, 5 μm), XBridge (50 × 4.6 mm, 5 μm), Gemini NX-C18 (50 × 4.6, 3 μm), or Gemini NX-C18 (50 × 4.6, 5 μm). A flow rate of 1.0 to 1.2 ml/min was used with mobile phase A: water + 0.1% modifier (v/v) and phase B: acetonitrile + 0.1% modifier (v/v). For acidic runs, the modifier was trifluoroacetic acid. A Shimadzu 20AB pump ran a gradient elution from 0 to 98% B over 2 min followed by a 1-min hold at 95% B. Detection was achieved using a Shimadzu 10A detector set at 220 or 260 nm followed in series by a Shimadzu MS 2010 EV or Applied Biosystems API 2000 mass spectrometer in parallel. The Shimadzu MS 2010 EV was tuned with the following parameters: 
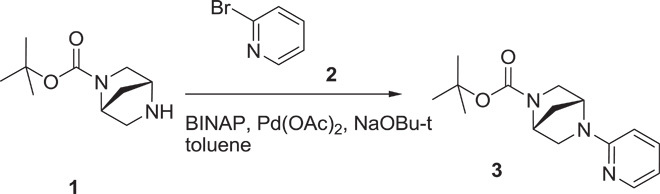
(1*R*,4*R*)-*tert*-butyl 5-(pyridin-2-yl)-2,5-diazabicyclo[2.2.1]heptane-2-carboxylate (**3**): To a stirred mixture of (1*R*,4*R*)-*tert*-butyl 2,5-diazabicyclo[2.2.1]heptane-2-carboxylate {500 mg, 2.52 mmol, [α]^20^ = 49.8° (*c* = 0.79 EtOH)} in toluene (~12.6 ml), 2-bromopyridine (0.481 ml, 5.04 mmol), potassium, *tert*-butoxide (1 M in THF, 2.52 ml, 2.52 mmol), palladium acetate (28 mg, 0.126 mmol), and BINAP (78 mg, 0.126 mmol) were added at room temperature, and then stirred for another 16 hours at 110°C. Thin-layer chromatography (TLC) (DCM/MeOH = 10:1) showed the reaction was complete. Water (3 ml) was added, the mixture was extracted with EA (10 × 3), and the organic layer was dried by anhydrous Na_2_SO_4_ and purified by column chromatography (50% petroleum ether in ethyl acetate) to give **3** as a yellow solid (620 mg, 89%). ^1^H NMR (400 MHz, DMSO-d_6_) δ = 1.31 to 1.46 (m, 9 H), 1.90 (d, *J* = 11.32 Hz, 1 H), 3.18 (d, *J* = 9.76 Hz, 1 H), 3.25 (d, *J* = 9.37 Hz, 1 H), 3.29 to 3.40 (m, 1 H), 3.49 (t, *J* = 8.39 Hz, 1 H), 4.39 to 4.54 (m, 1 H), 4.78 (d, *J* = 6.63 Hz, 1 H), 6.49 to 6.63 (m, 1 H), 7.46 to 7.56 (m, 1 H), 8.07 (d, *J* = 5.07 Hz, 1 H). 
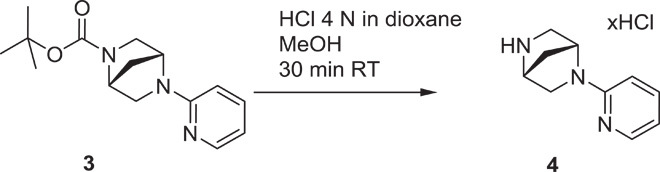
(1*R*,4*R*)-2-(pyridin-2-yl)-2,5-diazabicyclo[2.2.1]heptanes (**4**): To a solution of the starting material **3** (620 mg, 2.25 mmol, 1.0 eq) in methanol (1 ml), 4 N HCl in dioxane (5 ml, 20 mmol, 9 eq) was added. The reaction was homogeneous and slightly yellow in color. The reaction was stirred for 30 min, and LCMS/TLC indicated the consumption of the starting material and a new peak with the correct M + H (=176). The reaction was concentrated to a yellow solid and used without further purification as the HCl salt. LCMS = 90%, *t* = 0.16, mass/charge ratio (*m/z*) = 176.0. 
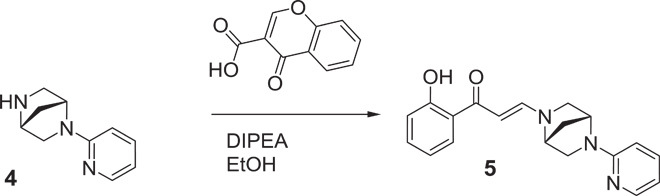
(*E*)-1-(2-hydroxyphenyl)-3-((1*R*,4*R*)-5-(pyridin-2-yl)-2,5-diazabicyclo[2.2.1]heptan-2-yl)prop-2-en-1-one (**5**): To a solution of the starting material amine **4** (477 mg, 2.25 mmol, 1.0 eq) in ethanol (11.3 ml), di-isopropylethylamine (1.98 ml, 11.3 mmol, 5.0 eq) was added. The reaction was stirred and was homogeneous. To the reaction, chromone-3-carboxylic acid (428 mg, 2.25, 1.0 eq) was added. The reaction was stirred at room temperature and followed by LCMS and TLC. The reaction was judged complete after 3 hours, and the reaction was concentrated to an oil residue. The residue was purified via a silica column (80 g, 20% ethyl acetate in heptane to 100% over 10 CV; desired material started eluting at ~80% ethyl acetate and peaked at ~85% ethyl acetate) to provide the desired material as a yellow solid (620 mg, 86%). ^1^H NMR (400 MHz, DMSO-d_6_) δ = 2.02 to 2.12 (m, 2 H), 3.34 to 3.41 (m, 2 H), 3.53 (d, *J* = 11.32 Hz, 1 H), 3.62 (d, *J* = 8.98 Hz, 1 H), 4.80 (s, 1 H), 4.98 (s, 1 H), 5.65 to 5.90 (m, 2 H), 6.50 to 6.66 (m, 2 H), 6.76 to 6.83 (m, 2 H), 7.35 (t, *J* = 7.61 Hz, 1 H), 7.49 to 7.59 (m, 1 H), 7.83 to 7.90 (m, 1 H), 8.10 (d, *J* = 4.68 Hz, 1 H), 8.26 (d, *J* = 12.10 Hz, 1 H); ^1^H NMR (400 MHz, chloroform-d) δ = 2.04 to 2.20 (m, 2 H), 3.35 to 3.55 (m, 3 H), 3.69 (d, *J* = 8.53 Hz, 1 H), 4.44 (br. s., 1 H), 5.08 (br. s., 1 H), 5.71 (d, *J* = 12.05 Hz, 1 H), 6.36 (d, *J* = 8.53 Hz, 1 H), 6.62 to 6.67 (m, 1 H), 6.79 (t, *J* = 7.78 Hz, 1 H), 6.92 (d, *J* = 7.53 Hz, 1 H), 7.34 (t, *J* = 7.03 Hz, 1 H), 7.48 (t, *J* = 8.28 Hz, 1 H), 7.61 (d, *J* = 9.03 Hz, 1 H), 8.09 to 8.20 (m, 2 H), 13.85 (s, 1 H); ^13^C NMR (DMSO-d_6_) δ = 36.9, 54.2, 55.6, 55.9, 63.9, 89.8, 107.2, 112,4, 117.4, 117.9, 119.9, 128.8, 133.8, 137.3, 147.9, 150.4, 156.9, 162.4, 189.9; LCMS = 100%, *t* = 0.60, *m/z* = 322.1; HRMS [M + H] for C_19_H_20_N_3_O_2_, calc., 322.1550, found, 322.1551; [α]^20^ = 120.5° (*c* = 0.22 MeOH).

### Protein crystallization

SMARCA2 construct (UniProt identifier SMCA2_HUMAN P51531-2 fragments 1373 to 1493) was used for the apo and cocrystal structures, and SMARCA4 construct (UniProt identifier SMCA4_ HUMAN P51532-1 fragments 1453 to 1569) was used for crystallographic study with the probe complex. Aliquots of the purified proteins were set up for crystallization using a mosquito crystallization robot (TTP Labtech). Coarse screens were typically set up onto Greiner three-well plates using three different drop ratios of precipitant to protein per condition (200 + 100, 150 + 150, and 100 + 200 nl). All crystallizations were carried out using the sitting drop vapor diffusion method at 4°C. SMARCA2 crystal of the free form was obtained by mixing 100 nl of the protein (21 mg/ml) and 200 nl of the crystallization buffer (15% PEG 6K, 8% ethylene glycol, 0.01 M ZnCl_2_). Crystals of SMARCA2 in complex with PFI-3 (2 mM final concentration) were obtained by mixing 200 nl of the protein (18 mg/ml) and 100 nl of the crystallization buffer (19% PEG 6K, 4% ethylene glycol, 0.01 M ZnCl_2_). Prism-like crystals of SMARCA4 with PFI-3 (3 mM final concentration) were grown by mixing 200 nl of the protein (18 mg/ml) and 100 nl of the crystallization buffer [0.1 M bis-tris propane (pH 7.2), 0.1 M ammonium acetate, 0.1 M ZnCl_2_, 15% PEG smear high].

### Data collection and structure solution

Crystals were cryoprotected using the well solution supplemented with an additional 20% ethylene glycol and were flash-frozen in liquid nitrogen. Data were collected at Diamond Light Source beamline I03 at a wavelength of 0.9763 Å. Indexing and integration were carried out using XDS (*39*), and scaling was performed with AIMLESS (*40*). Initial phases were calculated by molecular replacement with PHASER (*41*) using an ensemble of known bromodomain models (PDB IDs: 2OSS, 2OUO, 2GRC, 2OO1, 3DAI, 3D7C, 3DWY, and 3G0L). Unique and initial solutions were improved in a total of 50 cycles of automated protein chain tracing starting from existing model and computed using ARP/wARP (*42*). COOT was used for further manual building (*43*), and REFMAC5 for refinement against maximum likelihood target (*44*). Thermal motions were analyzed using TLSMD (translation/libration/screw motion determination), and hydrogen atoms were included in late refinement cycles. PRODRG (*45*) was used to generate compound coordinates and cif files. All model validations were carried out using MolProbity (*46*). Data collection and refinement statistics are compiled in table S3.

### Molecular dynamics

Molecular dynamics simulations of apo-PB1(5) and apo-BRD4(1) were based on the solved x-ray crystal structures. The simulation boxes were prepared using AmberTools14 (*47*), whereas molecular dynamics was performed in GROMACS 5.0 (*48*). The Amber ff14SB force filed was used for the proteins, and the TIP3P model was used for water molecules (*49*). All crystallographic water molecules were kept. The proteins were solvated and neutralized with chloride ions in a cubic box with periodic boundary conditions and a minimum distance between the solute and the box of 12 Å. A total of 10,000 energy minimization steps were carried out using a steepest descent algorithm. The system was equilibrated for 100 ps in the NVT and 200 ps in the NPT ensemble. Langevin dynamics was run with a reference temperature of 298.15 K, a time step of 2 fs, and the P-LINCS constraint algorithm (*50*) on atoms covalently bound to hydrogen atoms. The Berendsen weak coupling algorithm was used for NPT equilibration (*51*). Both systems were subsequently simulated for 50 ns in the NPT ensemble, saving coordinates every 0.1 ps, and using the Parrinello-Rahman pressure coupling scheme (*52*). Harmonic position restraints were applied to the solute heavy atoms with a force constant of 10 kcal mol^−1^ Å^−2^. The particle mesh Ewald algorithm was used for electrostatic interactions with a real space cutoff of 12 Å. A switch function between 9 and 10 Å was used for the Van der Waals interactions. The hydration thermodynamic properties of the two binding pockets were investigated using grid inhomogeneous solvation theory as implemented in AmberTools14 (*53*). Interaction energies between waters referred to as w1-4 in the text and the binding pocket residues of PB1(5) and BRD4(1) were analyzed using the python toolkit MDAnalysis (*54*) and the GROMACS tools. Four spherical hydration sites with 1.2-Å radius were defined and centered on the crystallographic water coordinates; for PB1(5), the water positions were taken from the crystal structure PDB-ID:3MB4 after structural alignment. Frames, in which all sites were occupied, forming the crystallographically observed network, were extracted, and the interaction energies between the waters and the binding pocket residues were obtained with *gmx energy*.

### Statistics

In vitro screening data were repeated at least three times, and average values as well as SEM are shown. In FRAP experiments, the fitted recovery curves represent averaged data of at least 10 replicates. The Student’s *t* test was used for comparisons of the mean values with wild-type treated cells and resulted in *P* ≤ 0.0001. qPCR experiments were independently repeated at least three times in triplicate (data in the figure had *P* < 0.05; *P* < 0.001, respectively). RNA-seq data analysis was based on at least 12 million reads for each of the replicates.

## SUPPLEMENTARY MATERIALS

Supplementary material for this article is available at http://advances.sciencemag.org/cgi/content/full/1/10/e1500723/DC1 Fig. S1. Sequence alignment and domain organization. Fig. S2. Acetyl lysine binding site comparisons. Fig. S3. Hydration thermodynamics of structural waters. Fig. S4. DNA intercalation data of PFI-3. Fig. S5. BLI data on the association of BRG1 with PFI-3. Fig. S6. Characterization of the negative control compound. Fig. S7. Stability of PFI-3 in cell culture medium. Fig. S8. Transcription and chromatin modification of Brg1 target genes are affected by PFI-3. Fig. S9. Gene set analysis. Fig. S10. TSC differentiation is affected by inhibition or knockdown of members of the SWI/SNF complex. Table S1. Temperature shift data for SA and PFI-3. Table S2. ITC data for SA and PFI-3. Table S3. Crystallographic data collection and refinement. Table S4. CEREP screening data (GPCRs and enzymes). Table S5. Significantly regulated genes. Table S6. Primers used in ChIP. Table S7. Primers used in qRT-PCR. Table S8. Sequences of used miRNA.
